# Minimally invasive video-assisted parathyroidectomy (MIVAP) versus conventional parathyroidectomy for renal hyperparathyroidism: a retrospective multicenter study

**DOI:** 10.1007/s13304-022-01291-9

**Published:** 2022-05-25

**Authors:** Iurii Snopok, Richard Viebahn, Martin Walz, Panagiota Zgoura, Pier Francesco Alesina

**Affiliations:** 1 Department of Surgery, Ruhr-University Bochum, Knappschaftskrankenhaus, Bochum, Germany; 2 Klinik Für Chirurgie and Zentrum Für Minimal Invasive Chirurgie, Evang, Kliniken Essen-Mitte, Essen, Germany; 3 Medical Department I, Ruhr-University Bochum, University Hospital Marienhospital Herne, Herne, Germany; 4 Klinik Für Endokrine Chirurgie, Helios University Hospital Wuppertal, Wuppertal, Germany

**Keywords:** MIVAP, Parathyroidectomy, Renal hyperparathyroidism, Renal insufficiency, Retrospective, Propensity score matching

## Abstract

To compare minimally invasive video-assisted parathyroidectomy (MIVAP) versus conventional surgery for renal hyperparathyroidism (rHPT). Between 2006 and 2020, 53 patients underwent MIVAP and 182 underwent conventional parathyroidectomy for rHPT at the Kliniken Essen-Mitte and Knappschaftskrankenhaus Bochum, respectively. Two propensity score-matched groups were retrospectively analyzed: the MIVAP group (VG; *n* = 53) and the conventional group (CG; *n* = 53). To assess long-term results, the patients were questioned prospectively (VG; *n* = 17, and CG; *n* = 26). The VG had a smaller incision (2.8 vs. 4.8 cm), shorter operation duration (81.0 vs. 13.9 min), and shorter duration of stay (2.4 vs. 5.7 days) (*p* < 0.0001) but a smaller drop in parathyroid hormone (PTH) postoperatively (81.3 vs. 85.5%. *p* = 0.022) than the CG. The conversion rate was 9.4% (*n* = 5). The VG had better Patient Scar Assessment Scale (PSAS) scores (10.8 vs. 11.7 *p* = 0.001) but worse SF-12 health survey scores (38.7 vs. 45.8 for physical health and 46.7 vs. 53.4 for mental health) (*p* < 0.0001). The PTH level at follow-up was higher in the VG (162.7 vs. 59.1 ng/l, *p* < 0.0001). There were no differences in morbidity, number of removed parathyroid glands, disease persistence, late rHPT relapse and need for repeat surgery between groups. MIVAP was superior to conventional parathyroidectomy regarding aesthetic outcomes and cost effectiveness. Conventional surgery showed better control of PTH levels and health scores on follow-up than MIVAP, without any impact on rHPT relapse and need for repeat surgery.

Trail registration number and date of registration: DRKS00022545 on 14.12.2020.

**Graphical abstract:**

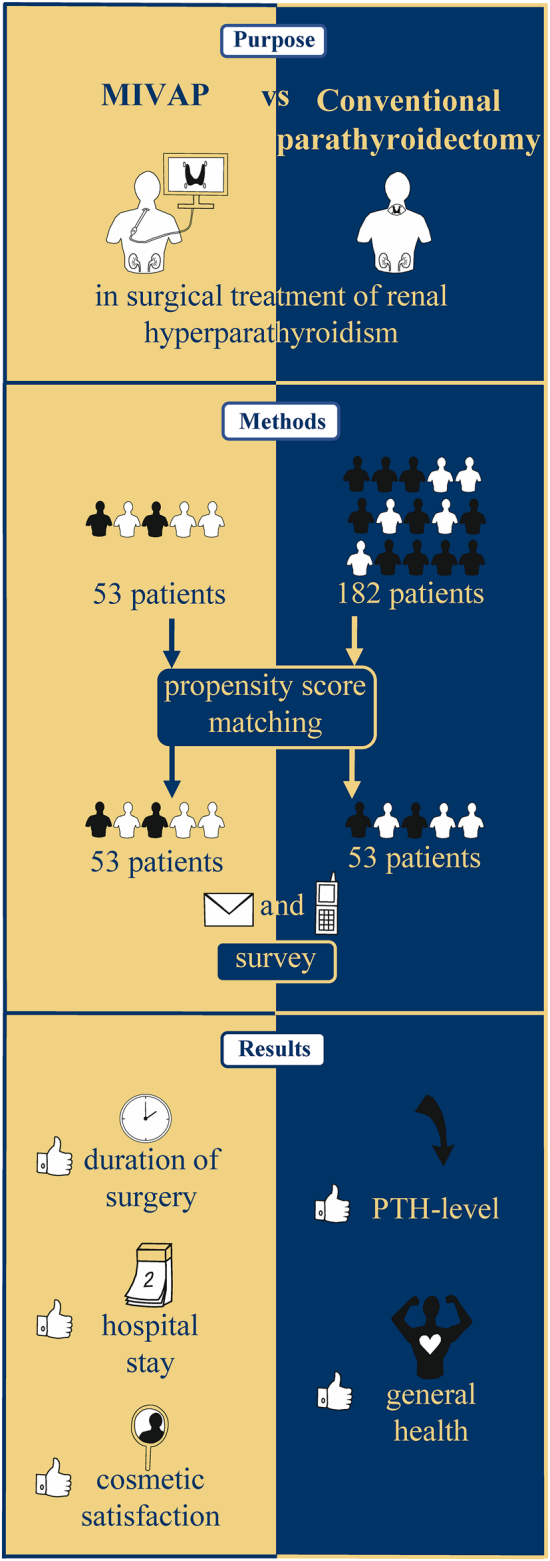

## Introduction

Renal hyperparathyroidism (rHPT) can occur as a result of terminal chronic kidney insufficiency and, if not treated, tends to be one of the main causes of morbidity and mortality in these patients. If properly indicated, surgery improves bone and cardiovascular status as well as survival in rHPT patients [1–3]. Bilateral neck exploration (BNE) through the Kocher collar incision with subtotal parathyroidectomy or total parathyroidectomy with or without autotransplantation of the parathyroid gland is a well-studied and standardized procedure for the treatment of rHPT [4–8]. Numerous minimally invasive procedures have been developed to treat primary hyperparathyroidism (pHPT) with a focused approach based on precise localization of the hyperplastic parathyroid gland and were eventually adapted for BNE in nonlocalized pHPT and rHPT [7, 9–19].

Minimally invasive video-assisted parathyroidectomy (MIVAP) appears to be the most reproducible method of minimally invasive parathyroid surgery with the same operative principles as open minimally invasive surgery (OMIP) and conventional BNE but utilizes a much smaller collar incision due to its video-assisted nature and shows better results in pHPT [20–23].

In contrast to studies on pHPT, only one small retrospective comparative study and a handful of case reports or small series describing the results of diverse minimally invasive video-assisted parathyroidectomy methods in rHPT are currently available. Only four of these papers focus on MIVAP, whereas the other five report pure endoscopic parathyroidectomy through different approaches [17, 21, 24–28]. The advantages and disadvantages of MIVAP compared to the reference procedure of conventional BNE with total or subtotal parathyroidectomy for the treatment of rHPT have not yet been studied. The small studies currently available have only suggested the feasibility and safety of MIVAP for rHPT, but not its efficacy profile during short- and long-term follow-up.

To the best of our knowledge, this is the first study to retrospectively compare the short- and long-term results of MIVAP versus conventional surgery in patients with rHPT from two surgical centers in Germany.

## Methods

### Study design

This retrospective study was designed to compare the safety, feasibility, and short- and long-term results of MIVAP (study group) versus conventional surgery (control group) for rHPT. The study protocol was approved by the ethics committee of the Ruhr-University Bochum and registered at the German Register of Clinical Trails (DRKS00022545).

**Fig. 1:**
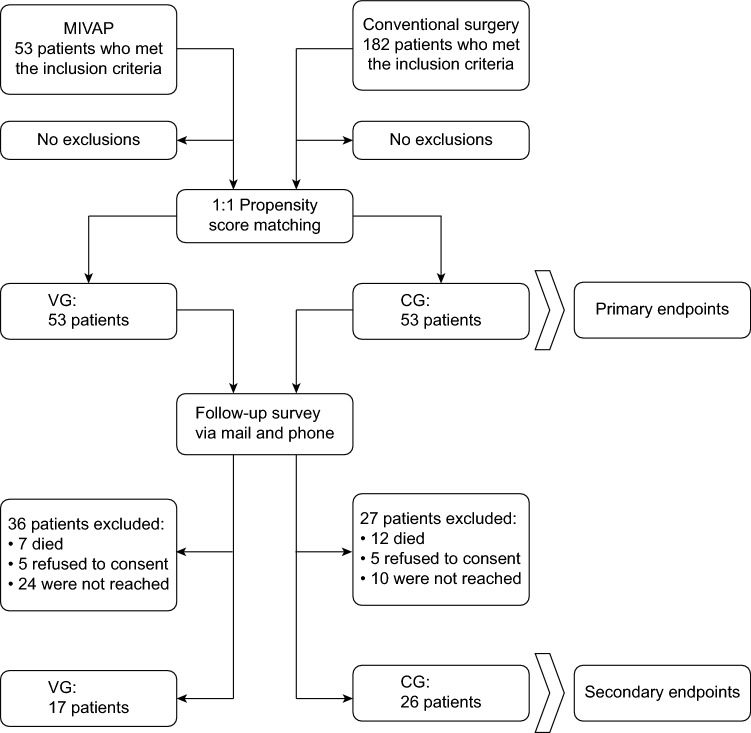
Patient’s flow of the study 53 patients at Kliniken Essen-Mitte and 182 patients at Knappschaftskrankenhaus Bochum met the inclusion criteria and were matched 1:1 to obtain 2 study groups, the MIVAP group (VG; *n* = 53) versus the conventional group (CG; *n* = 53), to analyze the primary endpoint data. For the secondary endpoint data, these patients were questioned via mail and phone (VG; *n = *17 and CG; *n* = 26)

All patients with rHPT who underwent MIVAP or conventional parathyroidectomy between January 2006 and July 2020 at Kliniken Essen Mitte or Knappschaftskrankenhaus Bochum, respectively, were included in the study. The exclusion criteria were patients with incomplete data in the electronic patient chart to extract primary endpoints, patients not reached follow-up and patients who did not consent to the follow-up survey. All patients who were contacted for surveillance provided consent to participate in the follow-up survey. We first identified all patients who met the inclusion criteria of the hospital information system in both centers. Then, the relevant data from the electronic patient charts of the included patients were extracted and coded in case report forms (CRFs). The data set was evaluated for missing data to exclude patients with incomplete data. Then, the patients in the MIVAP group and conventional group were matched with propensity score matching to obtain two comparable and equal study groups (MIVAP group—VG vs. conventional group—CG). Only the patients in these study groups were used for statistical analysis of the primary endpoints and were contacted via post and phone when obtaining data of the secondary endpoints. Figure 1 shows the patient flowchart of the study.

The primary endpoints were the duration of surgery, duration of hospital stay after surgery, morbidity and mortality, conversion rate for MIVAP, decrease in PTH level after surgery (expressed as % from the initial level), incision length, and number of removed parathyroid glands. The secondary endpoints were the Patient Scar Assessment Scale (PSAS) scores, SF-12 health survey scores, late recurrence rate of rHPT, rate of repeat surgery for rHPT relapse, last known PTH level on follow-up, correlation of incision length to the PSAS score and correlation of SF-12 scores to the PSAS score.

The PSAS was used to evaluate patient satisfaction with the aesthetic appearance of their scars. This scale contains six questions about the characteristics of the surgical scar, with each characteristic evaluated on a scale from 1 to 10, where a higher number of points corresponds to poorer satisfaction [29].

The SF-12-health survey was used to evaluate the patients’ opinion about their current health state based on 12 questions in 8 different fields and outputs points for physical and mental health. The higher the number of points is, the better the health status [30].

Late relapse of rHPT was defined as an elevation in PTH level > 500 ng/l on follow-up. Recurrent laryngeal nerve palsy was defined as dysfunction and clinical dysphonia that occurred postoperatively with or without signal alterations observed intraoperatively on neuromonitoring. Postoperative hypoparathyroidism was defined as a symptomatic drop in PTH level under 10 ng/l requiring intravenous calcium treatment. Persistent hyperparathyroidism was assumed when the PTH level did not drop by more than 50% of the initial level or did not return to normal range between 10 and 80 ng/l.

All patients underwent indirect laryngoscopy pre- and postoperatively. In all cases, intraoperative neuromonitoring and an intraoperative PTH level assessment were performed before preparation and 15 min after parathyroidectomy. MIVAP was performed with a modified Miccoli technique [16]. The patient’s head was not hyperextended. A central 2- to 3-cm skin incision was performed. In addition to the surgeon, two assistants were necessary: one holding the 30° 5-mm endoscope and the other providing retraction. After video-assisted exploration of all four glands, removal was started in the upper locations. Conventional surgery was performed by reclining the patient’s head through a 4–6 cm Kocher cervicotomy to explore all four parathyroid glands. The extent of the surgery in both groups was bilateral neck exploration with subtotal parathyroidectomy and transcervical thymus resection. If possible, one of the caudal parathyroid glands with the most normal macroscopic appearance was reduced by 50%, labeled with a titan clip, and preserved in situ. Redon drains were always placed after conventional surgery and selectively after MIVAP.

### Statistics and data presentation

XLSTAT Add-In for Microsoft Excel (Version 2021.1.1. Produced by: Addinsoft 2021, New York, USA) was utilized for statistical analysis.

In this study, 1:1 propensity score matching was performed using the greedy algorithm with Mahalanobis distance as a matching method with a caliper of 0.1 × Sigma, confidence interval of 99% and tolerance of 0.001 to obtain comparable study groups based on the following variables: age, sex, PTH level before surgery, year of surgery, diagnosis (secondary or tertiary HPT and relapse of secondary or tertiary HPT) and anamnesis (dialysis, renal transplant, other health conditions and combinations of these). The numerical data were evaluated using the Mann–Whitney *U* test. Categorical data were evaluated using the chi-square/Fisher exact test. Correlations were analyzed using Spearman’s correlation coefficient. A *p* value < 0.05 was considered significant.

The numeric data are presented as means and standard deviations of the mean. The categoric data are presented as percentages.

## Results

### Propensity score matching

The comparison of the variables used for propensity score matching between the matched and unmatched patient groups showed higher *p* values in the matched groups, suggesting higher similarity and thus better comparability (Table 1).

**Table 1:** Comparison of patient variables before and after propensity score matching (1: 1), demonstrating the higher similarity (higher *p* values) between study groups with matched patients

Variable	Unmatched patients		*p* value	Matched patients		*p* value
	VG (*n* = 53)	CG (*n* = 182)		VG (*n* = 53)	CG *(n* = 53)	
Age, years (mean ± SD)	49.9 ± 14.7	52.4 ± 13.5	0.238	49.9 ± 14.7	50.0 ± 15.1	0.964
Preoperative PTH, ng/l (mean ± SD)	966.3 ± 634.1	995.9 ± 632.7	0.643	966.3 ± 634.1	954.2 ± 643.9	0.884
Sex, *n* (%)			0.272			0.846
Female	29 (54.7)	84 (46.2)		29 (54.7)	28 (52.8)	
Male	24 (45.3)	98 (53.8)		24 (45.3)	25 (47.2)	
Diagnosis, *n* (%)			0.47			1
sHPT	49 (92.5)	159 (87.4)		49 (92.5)	48 (90.6)	
tHPT	3 (5.7)	14 (7.7)		3 (5.7)	4 (7.6)	
sHPT relapse	0 (0)	7 (3.9)		0 (0)	0 (0)	
tHPT relapse	1 (1.9)	2 (1.1)		1 (1.9)	1 (1.9)	
Anamnesis, *n* (%):			0.425			0.763
Dialysis	45 (84.9)	146 (80.2)		45 (84.9)	40 (75.5)	
Hospitalization after a kidney transplant	5 (9.4)	29 (15.9)		5 (9.4)	9 (17.0)	
Other relevant conditions	2 (3.8)	6 (3.3)		2 (3.8)	3 (5.7)	
Dialysis and other relevant conditions	1 (1.9)	1 (0.6)		1 (1.9)	1 (1.9)	
Year of surgery, *n* (%):			0.007			0.997
2006	2 (3.8)	13 (7.1)		2 (3.8)	4 (7.6)	
2007	3 (5.7)	15 (8.2)		3 (5.7)	2 (3.8)	
2008	9 (17.0)	14 (7.7)		9 (17.0)	13 (24.5)	
2009	5 (9.4)	8 (4.4)		5 (9.4)	3 (5.7)	
2010	5 (9.4)	5 (2.8)		5 (9.4)	4 (7.6)	
2011	4 (7.6)	3 (1.7)		4 (7.6)	3 (5.7)	
2012	4 (7.6)	8 (4.4)		4 (7.6)	5 (9.4)	
2013	4 (7.6)	4 (2.2)		4 (7.6)	4 (7.6)	
2014	6 (11.3)	29 (15.9)		6 (11.3)	3 (5.7)	
2015	3 (5.7)	18 (9.9)		3 (5.7)	3 (5.7)	
2016	1 (1.9)	16 (8.8)		1 (1.9)	1 (1.9)	
2017	1 (1.9)	15 (8.2)		1 (1.9)	1 (1.9)	
2018	2 (3.8)	21 (11.5)		2 (3.8)	3 (5.7)	
2019	2 (3.8)	10 (5.5)		2 (3.8)	2 (3.8)	
2020	2 (3.8)	3 (1.7)		2 (3.8)	2 (3.8)	

*PTH* parathormone, *sHPT* secondary hyperparathyroidism, *tHPT* tertiary hyperparathyroidism, *VG* MIVAP group, *CV* conventional group, *SD* standard deviation of the mean

### Primary endpoints

The conversion rate to open surgery was 9.4%. The VG showed a shorter operation duration and hospital stay and a smaller skin incision but a smaller drop in PTH levels postoperatively than the CG (Table 2).

**Table 2:** Comparison of short-term outcomes after surgery

Variable	VG (*n* = 53)	CG (*n* = 53)	*p* value
Duration of surgery, min (mean ± SD)	81.0 ± 38.2	133.9 ± 51.6	< 0.0001**
Duration of hospital stay, days (mean ± SD)	2.4 ± 0.8	5.7 ± 4.4	< 0.0001**
Mortality, *n* (%)	0 (0)	0 (0)	–
Complications, *n* (%):			
Overall	13 (24.5)	11 (20.8)	0.817
Recurrent laryngeal nerve palsy	2 (3.8)	4 (7.6)	0.357
Hypocalcemia/hypoparathyroidism	7 (13.2)	3 (5.7)	0.24
Bleeding	0 (0)	2 (3.8)	0.199
Persistent HPT	3 (5.7)	2 (3.8)	1.0
Others	1 (1.9)*	0 (0)	1.0
Conversion to open surgery, *n* (%)	5 (9.4)	0 (0)	–
Decrease in PTH level after surgery, % (mean ± SD)	81.3 ± 15.0	85.5 ± 16.1	0.022**
Length of incision, cm (mean ± SD)	2.8 ± 0.9	4.8 ± 1.3	< 0.0001**
Number of removed glands, *n* (mean ± SD)	3.6 ± 0.8	3.5 ± 0.9	0.936

*PTH* parathormone, *HPT* hyperparathyroidism, *VG* MIVAP group, *CV* conventional group, *SD* standard deviation of the mean

*Arrhythmia due to intraoperative hyponatremia

**Statistically significant

### Secondary endpoints

**Fig. 2:**
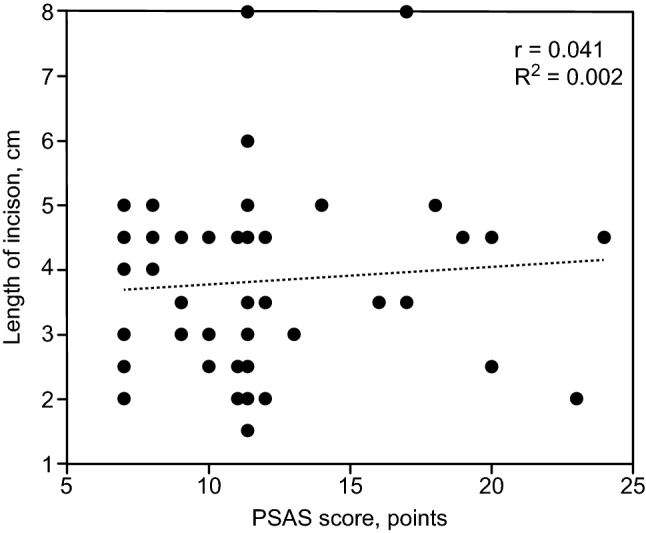
Correlation between length of incision and PSAS score. The correlation between the length of the incision and PSAS score in patients in both study groups pooled together, as assessed using Spearman’s correlation coefficient (*r*) and coefficient of determination (*R*^2^). A weak nonsignificant positive correlation (*r* = 0.041, *p* = 0.678, *R*^2^ = 0.002) was shown

**Fig. 3:**
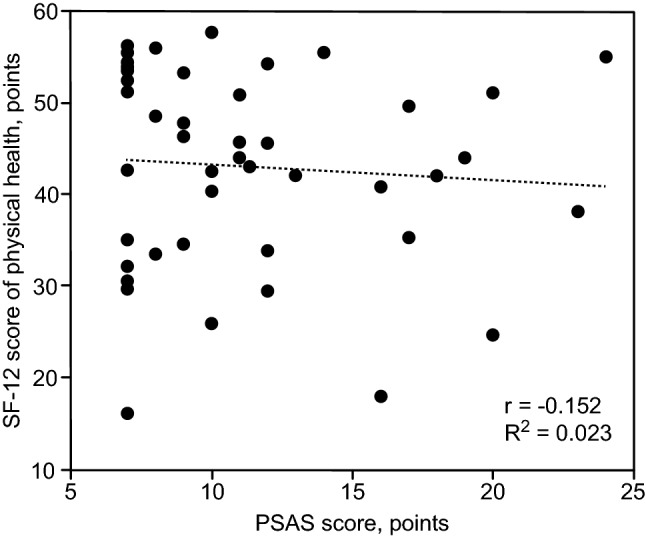
Correlation between the physical health SF-12 score and the PSAS score The correlation between the SF-12 health survey scores of physical health and the PASA score of patients in both study groups pooled together was assessed using Spearman’s correlation coefficient (*r*) and coefficient of determination (*R*^2^). A weak nonsignificant negative correlation (*r *= − 0.152, *p *= 0.119, *R*^2^ = 0.023) was shown

The mean follow-up period was 93 months (range from 14 to 198 months) for both study groups. The VG showed better (lower) PSAS scores but poorer SF-12 health survey scores for both for physical and mental health as well as higher last known PTH levels at follow-up than the CG (Table 3). There was no clinically or statistically significant correlation between the length of the incision and the PSAS score or between the SF-12 scores and PSAS scores in either study group (Figs. 2 and 3).

**Table 3:** Comparison of long-term outcomes at follow-up

Variable	VG (*n* = 17)	CG (*n* = 26)	*p* value
PSAS score, points (mean ± SD)	10.8 ± 2.5	11.7 ± 3.5	0.001*
SF-12 score of physical health, points (mean ± SD)	38.7 ± 5.5	45.8 ± 7.6	< 0.0001*
SF-12 score of mental health, points (mean ± SD)	46.7 ± 5.4	53.4 ± 5.9	< 0.0001*
Late relapse of rHPT, *n* (%)	2 (11.8)	2 (7.7)	1
Repeated surgery for relapse, *n* (%)	1 (5.9)	2 (7.7)	1
PTH level on follow-up, ng/l (mean ± SD)	162.7 ± 65.9	59.1 ± 41.9	< 0.0001*

*PSAS* Patient Scar Assessment Scale, *SF*-12 The Short Form (12) Health Survey, *PTH* parathormone, *HPT* hyperparathyroidism, *VG* MIVAP group, *CV* conventional group, *SD* standard deviation of the mean

*Statistically significant

The primary endpoint results have more statistical power, since they were obtained from the larger patient population (*n* = 106), compared to population analyzed for the secondary endpoints (*n* = 43).

## Discussion

This study is the first retrospective comparison of MIVAP vs. conventional surgery in patients with rHPT and confirmed the feasibility and safety of MIVAP by showing long- and short-term outcomes that mostly resemble, and at some points outperform, those of conventional surgery.

Our results suggest the feasibility of MIVAP, with an acceptable conversion rate of 9.4%, and no difference in the number of removed parathyroid glands compared to conventional surgery. There were also no significant differences in safety parameters (morbidity and mortality). MIVAP appeared to be superior to conventional parathyroidectomy in terms of aesthetic results (smaller incision, better PSAS score) and cost effectiveness (shorter operation duration and hospital stay). Conventional surgery showed better short- and long-term control of PTH levels and better SF-12 health scores on follow-up. These findings do not appear to be associated with an increase in persistent rHPT, rHPT relapse rate or need for repeated surgery in the MIVAP group.

We defined the conversion rate and number of removed parathyroid glands as parameters of feasibility. The conversion rate of 9.4% in this study is noticeably lower than that reported in previous studies of MIVAP for rHPT (0–30%) and therefore is acceptable [21, 25]. We suggest that the conversion rate would decrease with increasing experience in MIVAP. The number of removed parathyroid glands in the MIVAP group did not differ from that in the conventional surgery study, confirming the feasibility of MIVAP for rHPT [21, 25]. The number of approximately 3.5 in both groups corresponds to subtotal parathyroidectomy with transcervical thymus resection the best surgical strategy for patients currently listed for kidney transplantation [7, 31]. The number of removed glands also plays an important role in predicting postoperative disease persistence and late recurrence of rHPT (see below) [31].

The safety of the procedure is predominantly defined by its mortality and morbidity. There were no mortalities in either study group. The overall morbidity was 24.5% in the MIVAP group and 20.8% in the conventional group, showing no statistically significant difference. In comparison, Barbaros et al. [25] reported a 16.7% overall complication rate for MIVAP in rHPT and zero mortalities. The overall morbidity after conventional surgery for rHPT considering bleeding, recurrent laryngeal nerve palsy and wound infection according to current literature is under 5% [32]. Our morbidity results are higher than those in the literature, but considering only these three complications, our study would have shown overall complication rates of 3.8% for MIVAP and 11.3% for conventional surgery, which would not be significantly different from the published value. The currently reported rate of bleeding after surgery for rHPT is between 0.5 and 4.0%, which agrees with our results (Table 2) [7]. The rate of postoperative recurrent laryngeal nerve palsy in our study is similar that in the literature (1.3–10.5%), although we did not investigate the rate of late permanent recurrent laryngeal nerve palsy [5, 7]. There were no wound infections in our study.

Our results suggest that MIVAP may be more cost effective than conventional surgery due to the shorter operative time (< 50 min on average) and hospital stay (< 3 days on average). The material costs of MIVAP consist of expenses for the dedicated instrumentarium, which is reusable, and conventional laparoscopy; these may easily be compensated for by savings in personnel costs from surgery and the hospital stay.

The most important parameter of efficacy for rHPT surgery is the short- and long-term control of PTH levels. Our results show a significantly stronger decrease in PTH levels after conventional surgery than after MIVAP. We consider that this difference is not clinically relevant because the mean PTH drop after both techniques was over 70%, which corresponds to the Miami criteria as well as other widely accepted predictors of efficacy of a 60–70% PTH decrease [8]. Nevertheless, the larger PTH drop after conventional surgery may confirm that this approach remains the gold standard in the surgical treatment of rHPT. The rate of persistent rHPT in this study was 5.7% after MIVAP and 3.8% after conventional surgery, which was not a significant difference. This agrees with the reported rates of 3.6–22% for rate of persistent rHPT postoperatively [33–36]. The most likely cause of rHPT persistence after surgery is the supernumerary or ectopic parathyroid gland [31]. The PTH levels at follow-up were significantly higher in the MIVAP group. This was accompanied by a nonsignificantly higher (11.8%) rate of late rHPT recurrence after MIVAP than after conventional surgery (7.7%). The rate of repeated surgery due to recurrence was 5.9% in the MIVAP group vs. 7.7% after conventional surgery, which was also not a significant difference. In the current literature, the rate of late recurrence of rHPT after surgery is 4–9.5%, which is similar to our results [33–36]. The most reasonable cause of late relapses is hypertrophy of the preserved parathyroid gland.

The higher SF-12 health scores on follow-up in the conventional group may be partially explained by better control of the PTH levels. Interestingly, the PSAS score showed a weak negative correlation with SF-12 scores (Fig. 3). Although the correlation was not significant in our study, it appears logical that the patients with better health tended to be more satisfied in general as well as with, for example, the aesthetic outcomes of surgery.

We considered the length of surgical incision and PSAS score as parameters of aesthetic satisfaction. The length of the skin incision was 2.4 ± 0.12 cm in the study by Mourad et al. [21] and 2.7 ± 0.2 cm in the study of Barbaros et al. [25]; these are comparable with the incision in our MIVAP group. The significantly shorter skin incision in the MIVAP group may contribute to higher aesthetic satisfaction among these patients. The PSAS scores in the MIVAP group were significantly lower than in the conventional surgery group; thus, satisfaction with the aesthetic appearance of the scar was higher. To underpin this statement, the correlation coefficient between the length of the skin incision and PSAS score was calculated (Fig. 2).

Our study has two major limitations. MIVAP was performed exclusively in Kliniken Essen Mitte, and conventional surgery was performed exclusively in Knappschaftskrankenhaus Bochum. This could lead to selection bias that affects the study results because of differences in surgical teams and workflows in these clinics that cannot be equalized with propensity score matching. Sampling error is possible because of the small number of patients followed up, and the statistical power of the secondary endpoint analysis may be low. However, this is the largest population with rHPT to be evaluated in the current literature.

## Conclusions

MIVAP is feasible, safe, and effective for the treatment of rHPT, and it appears to be superior to conventional parathyroidectomy in terms of aesthetics, operation duration and length of hospital stay. Conventional surgery showed better short- and long-term control of PTH levels and better health scores on follow-up, without any impact on persistent rHPT, late recurrence of rHPT or need for repeated surgery, compared to MIVAP.

These results may motivate the wide adoption of MIVAP for the treatment of rHPT in the clinic. The cost-effectiveness data of MIVAP for rHPT seem to be interesting for further, cost-focused studies. The comparison of MIVAP with conventional surgery in rHPT is an attractive and ethically acceptable focus for randomized control trials.

## Data Availability

The deidentified data analyzed in this study are available upon reasonable request.

## References

[1] Van Der Plas WY, Dulfer RR, Koh EY (2018). Safety and efficacy of subtotal or total parathyroidectomy for patients with secondary or tertiary hyperparathyroidism in four academic centers in the Netherlands. Langenbecks Arch Surg.

[2] Ivarsson KM, Akaberi S, Isaksson E, Reihnér E, Czuba T, Prütz KG, Clyne N, Almquist M (2019). Cardiovascular and cerebrovascular events after parathyroidectomy in patients on renal replacement therapy. World J Surg.

[3] Chen L, Wang K, Yu S, Lai L, Zhang X, Yuan J, Duan W (2016). Long-term mortality after parathyroidectomy among chronic kidney disease patients with secondary hyperparathyroidism: a systematic review and meta-analysis. Ren Fail.

[4] Chen J, Jia X, Kong X, Wang Z, Cui M, Xu D (2017). Total parathyroidectomy with autotransplantation versus subtotal parathyroidectomy for renal hyperparathyroidism: a systematic review and meta-analysis. Nephrology (Carlton).

[5] Schlosser K, Bartsch DK, Diener MK, Seiler CM, Bruckner T, Nies C, Meyer M, Neudecker J, Goretzki PE, Glockzin G, Konopke R, Rothmund M (2016). Total parathyroidectomy with routine thymectomy and autotransplantation versus total parathyroidectomy alone for secondary hyperparathyroidism: results of a nonconfirmatory multicenter prospective randomized controlled pilot trial. Ann Surg.

[6] Liu ME, Qiu NC, Zha SL, Du ZP, Wang YF, Wang Q, Chen Q, Cen XX, Jiang Y, Luo Q, Shan CX, Qiu M (2017). To assess the effects of parathyroidectomy (TPTX versus TPTX + AT) for secondary hyperparathyroidism in chronic renal failure: a systematic review and meta-analysis. Int J Surg.

[7] Weber T, Dotzenrath C, Dralle H (2021). Management of primary and renal hyperparathyroidism: guidelines from the German association of endocrine surgeons (CAEK). Langenbecks Arch Surg.

[8] Steinl GK, Kuo JH (2021). Surgical management of secondary hyperparathyroidism. Kidney Int Rep.

[9] Roosli C, Bortoluzzi L, Linder TE, Muller W (2009). Role of minimal invasive surgery for primary and secondary hyperparathyroidism. Laryngorhinootologie.

[10] Norman J, Chheda H, Farrell C (1998). Minimally invasive parathyroidectomy for primary hyperparathyroidism: decreasing operative time and potential complications while improving cosmetic results. Am Surg.

[11] Udelsman R, Donovan PI, Sokoll LJ (2000). One hundred consecutive minimally invasive parathyroid explorations. Ann Surg.

[12] Agarwal G, Barraclough BH, Robinson BG, Reeve TS, Delbridge LW (2002). Minimally invasive parathyroidectomy using the ‘focused’ lateral approach. I. Results of the first 100 consecutive cases. ANZ J Surg.

[13] Gagner M (1996). Endoscopic subtotal parathyroidectomy in patients with primary hyperparathyroidism. Br J Surg.

[14] Bhandarwar A, Gala J, Arora E, Gajbhiye R, Talwar G, Gandhi S, Wagh A, Patel C (2021). Endoscopic parathyroidectomy: a retrospective review of 27 cases. Surg Endosc.

[15] Henry JF, Defechereux T, Gramatica L, De Boissezon C (1999). Minimally invasive videoscopic parathyroidectomy by lateral approach. Langenbecks Arch Surg.

[16] Miccoli P, Bendinelli C, Conte M, Pinchera A, Marcocci C (1998). Endoscopic parathyroidectomy by a gasless approach. J Laparoendosc Adv Surg Tech A.

[17] Sasanakietkul T, Jitpratoom P, Anuwong A (2017). Transoral endoscopic parathyroidectomy vestibular approach: a novel scarless parathyroid surgery. Surg Endosc.

[18] Wu YJ, Cheng BC, Chiu CH (2019). Successful modified transoral endoscopic parathyroidectomy vestibular approach for secondary hyperparathyroidism with ectopic mediastinal glands. Surg Laparosc Endosc Percutan Tech.

[19] Entezami P, Boven L, Ware E, Chang BA (2021). Transoral endoscopic parathyroidectomy vestibular approach: a systematic review. Am J Otolaryngol.

[20] Lorenz K, Miccoli P, Monchik JM, Düren M, Dralle H (2001). Minimally invasive video-assisted parathyroidectomy: multiinstitutional study. World J Surg.

[21] Mourad M, Ngongang C, Saab N, Coche E, Jamar F, Michel JM, Maiter D, Malaise J, Squifflet JP (2001). Video-assisted neck exploration for primary and secondary hyperparathyroidism: initial experience. Surg Endosc.

[22] Rulli F, Galata G, Pompeo E, Farinon AM (2007). A camera handler for Miccoli’s minimally invasive video-assisted thyroidectomy and paratiroidectomy procedures. Surg Endosc.

[23] Lombardi CP, Raffaelli M, Traini E, De Crea C, Corsello SM, Bellantone R (2009). Video-assisted minimally invasive parathyroidectomy: benefits and long-term results. World J Surg.

[24] Prosst RL, Gahlen J, Schnuelle P, Post S, Willeke F (2006). Fluorescence-guided minimally invasive parathyroidectomy: a novel surgical therapy for secondary hyperparathyroidism. Am J Kidney Dis.

[25] Barbaros U, Erbil Y, Yildirim A, Saricam G, Yazici H, Ozarmagan S (2009). Minimally invasive video-assisted subtotal parathyroidectomy with thymectomy for secondary hyperparathyroidism. Langenbecks Arch Surg.

[26] Alesina PF, Hinrichs J, Kribben A, Walz MK (2010). Minimally invasive video-assisted parathyroidectomy (MIVAP) for secondary hyperparathyroidism: report of initial experience. Am J Surg.

[27] Ikeda Y, Takami H, Niimi M, Kan S, Sasaki Y, Takayama J (2002). Endoscopic total parathyroidectomy by the anterior chest approach for renal hyperparathyroidism. Surg Endosc.

[28] He Q, Zhu J, Zhuang D, Fan Z (2015). Robotic total parathyroidectomy by the axillo-bilateral-breast approach for secondary hyperparathyroidism: a feasibility study. J Laparoendosc Adv Surg Tech A.

[29] Draaijers LJ, Tempelman FR, Botman YA, Tuinebreijer WE, Middelkoop E, Kreis RW, Van Zuijlen PP (2004). The patient and observer scar assessment scale: a reliable and feasible tool for scar evaluation. Plast Reconstr Surg.

[30] Ware J, Kosinski M, Keller SD (1996). A 12-item short-form health survey: construction of scales and preliminary tests of reliability and validity. Med Care.

[31] Reitz RJ, Dreimiller A, Khil A, Horwitz E, McHenry CR (2021). Ectopic and supernumerary parathyroid glands in patients with refractory renal hyperparathyroidism. Surgery.

[32] Nastos K, Constantinides V, Mizamtsidi M, Duncan N, Tolley N, Palazzo F (2018). Morbidity in parathyroid surgery for renal disease is under reported: a comparison of outcomes with primary hyperparathyroidism. Ann R Coll Surg Engl.

[33] Van Der Plas W, Kruijff S, Sidhu SB, Delbridge LW, Sywak MS, Engelsman AF (2021). Parathyroidectomy for patients with secondary hyperparathyroidism in a changing landscape for the management of end-stage renal disease. Surgery.

[34] Steffen L, Moffa G, Müller PC, Oertli D (2019). Secondary hyperparathyroidism: recurrence after total parathyroidectomy with autotransplantation. Swiss Med Wkly.

[35] Sari R, Yabanoglu H, Hargura AS, Kus M, Arer IM (2020). Outcomes of total parathyroidectomy with autotransplantation versus subtotal parathyroidectomy techniques for secondary hyperparathyroidism in chronic renal failure. J Coll Physicians Surg Pak.

[36] Choi HR, Aboueisha MA, Attia AS, Omar M, ELnahla A, Toraih EA, Shama M, Chung WY, Jeong JJ, Kandil E (2021). Outcomes of subtotal parathyroidectomy versus total parathyroidectomy with autotransplantation for tertiary hyperparathyroidism: multi-institutional study. Ann Surg.

